# Gap Junction Beta-2 p.Val84Met Can Cause Autosomal Dominant Syndromic Hearing Loss With Keratoderma

**DOI:** 10.7759/cureus.54992

**Published:** 2024-02-26

**Authors:** Kosuke Hashimoto, Toru Miwa, Chie Ono, Kiyomitsu Nara, Hideki Mutai, Toshiyuki Seto, Hirokazu Sakamoto, Tatsuo Matsunaga

**Affiliations:** 1 Department of Otolaryngology, Osaka Metropolitan University, Osaka, JPN; 2 Department of Otolaryngology, Head and Neck Surgery, Kyoto University, Kyoto, JPN; 3 Department of Clinical Genomics, Osaka Metropolitan University, Osaka, JPN; 4 Division of Hearing and Balance Research, National Institute of Sensory Organs, National Hospital Organization (NHO) Tokyo Medical Center, Tokyo, JPN

**Keywords:** keratoderma, missense variant, gjb2, connexin 26, syndromic hearing loss, autosomal dominant inheritance

## Abstract

In this study, we report a case of bilateral mild hearing loss and keratoderma caused by a gap junction beta-2 (GJB2) variant. The proband was a nine-year-old Japanese boy with bilateral mild hearing loss at birth. The proband’s father, sister, paternal aunt, and cousins had mild sensorineural hearing loss. Further evaluation revealed keratoderma on the feet of the proband, father, sister, paternal aunt, and cousins. We identified a heterozygous c.250G>A (p.Val84Met) variant in GJB2 as the cause of the autosomal dominant syndromic hearing loss with the skin disorder in this Japanese family and delineated the pathological significance of the variant. The Val84Met variant in GJB2 contributes to the autosomal dominant form of syndromic hearing loss with keratoderma.

## Introduction

One in every 1000 babies has severe-to-profound hearing loss, with hereditary factors accounting for over half of these cases. Autosomal recessive inheritance is more common than X-linked, autosomal dominant, and mitochondrial forms of inheritance. Pathogenic variants in the Gap junction beta-2 (GJB2) (NM_004004.5) gene are the most common cause of hereditary sensorineural deafness [1]. GJB2 encodes connexin 26 (Cx26), a member of the highly conserved gap junction protein family of intercellular channels that allow nearby cells to exchange molecules, ions, and electrical impulses. Each gap junction channel is formed by two linked hemichannels, termed connexons, one on each membrane of adjacent cells. Each connexon comprises a hexamer of the same or distinct connexin units.

Most reported GJB2 variants are associated with autosomal recessive non-syndromic hearing loss (ARNSHL) (Mendelian Inheritance in Man {MIM}: 220290). However, other variants are associated with autosomal dominant non-syndromic hearing loss (MIM: 601544) or autosomal dominant syndromic hearing loss (ADSHL) with differing cutaneous manifestations [2]. Syndromic hearing loss includes hystrix-like ichthyosis with deafness (MIM: 602540), Bart-Pumphrey syndrome (MIM: 149200), palmoplantar keratoderma with deafness (MIM: 148350), keratitis ichthyosis deafness syndrome (MIM:148210), and Vohwinkel syndrome (MIM: 124500), with a major overlap of clinical features. In general, congenital and non-syndromic hearing loss is caused by homozygous or compound heterozygous loss-of-function variants [3]. In contrast, gain-of-function variants may result in various degrees of hearing loss, which can occur independently or in conjunction with nail or skin conditions. This phenotypic variation may be caused by interactions between different connexins [2]. In addition, amino acid substitutions, which may change protein function, have been implicated in several diseases. Notably, most GJB2 variants disrupt normal connexin docking in the extracellular domain of Cx26 and are linked to either autosomal dominant syndromic or non-syndromic hearing loss clusters [1].

Most of the over 200 reported deafness-causing variants in GJB2 cause ARNSHL; however, over 30 GJB2 variants have been linked to autosomal dominant hearing loss (ADHL). Approximately two-thirds of all cases of ADHL include syndromic hearing loss with overt cutaneous manifestations, whereas the remaining third includes non-syndromic hearing loss. Thus, GJB2 variants drive diverse pathological phenotypes related to hearing and/or skin manifestations. Notably, to date, the GJB2 p.Val84Met variant has not been reported to cause syndromic hearing loss [4-7]. In this report, we present a novel case of the c.250G>A (p.Val84Met) variant causing ADSHL with keratoderma on the soles of the feet in a Japanese family.

## Case presentation

This study was approved by the Institutional Review Board of Osaka Metropolitan University (approval number: 3616) and conformed to the guidelines of The Declaration of Helsinki. Clinical assessment and genetic counseling were performed for each consenting person. All patients or their legal guardians (if the patients were under 20 years of age and wanted to participate in this study) provided informed consent.

**Figure 1:**
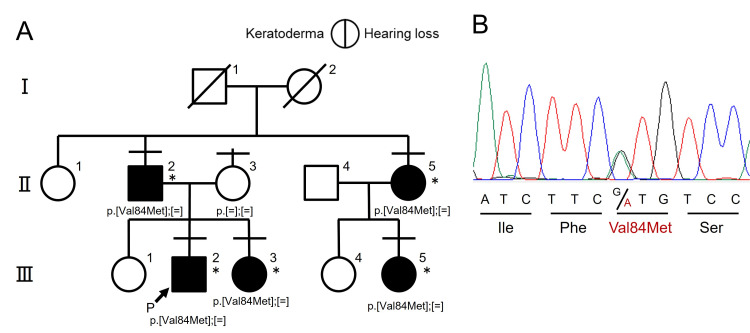
Family in this study. (A) Pedigree with genotypes of participants who underwent genetic analyses (crossbar above each individual). Asterisks show the clinical features evaluated. Proband is indicated by the capital letter P with an arrow. Square symbols, males; circle symbols, females; filled symbols on the right and left indicate hearing loss and skin abnormalities, respectively. (B) Partial electropherogram showing the heterozygous p.Val84Met variant. Image Credits: Kosuke Hashimoto and Hideki Mutai.

The proband was a nine-year-old boy (III-2, Figure 1). He was born following a normal pregnancy; however, he failed the newborn hearing screening in his left ear. Hearing loss was judged to be mild in the bilateral ears but not progressive. He was monitored by an otolaryngologist who performed hearing tests about once a year.

**Figure 2:**
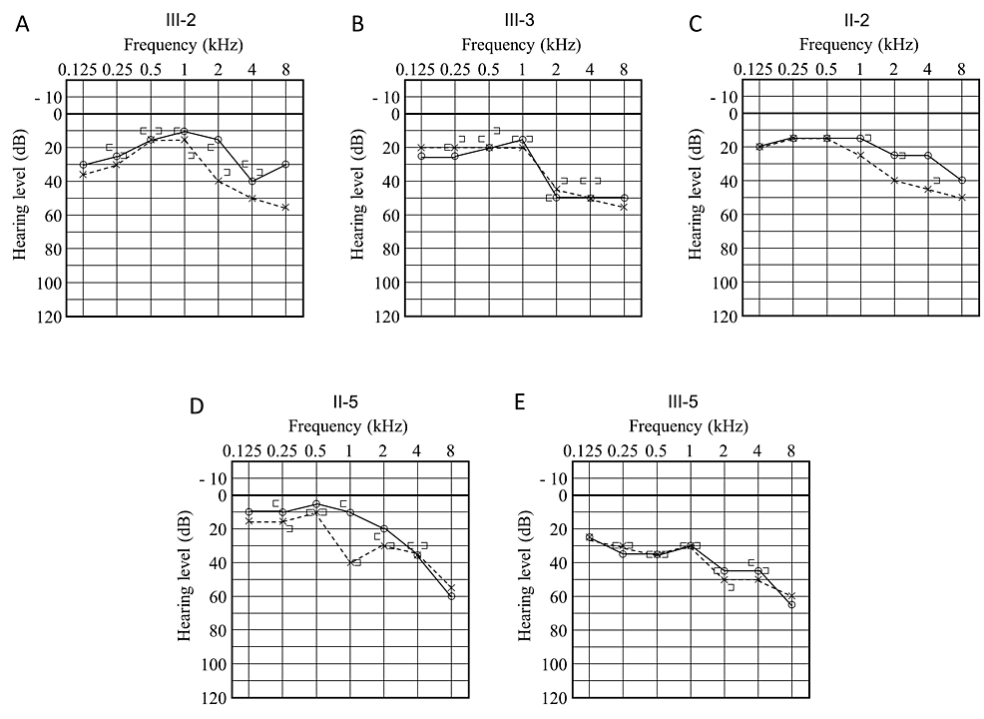
Audiograms of the patients. Audiograms of (A) proband (III-2), (B) III-3, (C) II-2, (D) II-5, and (E) III-5.

**Figure 3:**
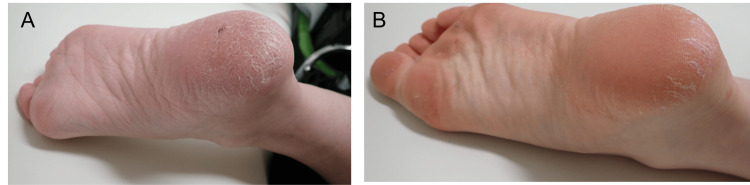
Keratoderma on the feet. Keratoderma on the left soles of (A) II-5 and (B) III-5.

At the age of nine years, the proband visited our department for genetic testing. Audiological evaluation revealed mild sensorineural hearing loss (SNHL) according to the GENDEAF criteria [8] (Figure 2A). Distortion-product otoacoustic emissions testing revealed a “pass” result in the right ear but a “refer” result in the left ear. The proband’s four-year-old sister (III-3) was also found to have bilateral mild SNHL (Figure 2B). She had failed the newborn hearing screening and had been using hearing aids in both ears since the age of three years. The father (II-2), aged 44 years, also had mild SNHL (Figure 2C). Keratoderma was also detected on the soles of the feet of the proband, father, and sister (data not shown), prompting further investigation of the clinical symptoms, including skin abnormalities in the other family members. The paternal aunt (II-5), aged 41 years, had mild SNHL along with keratoderma on the soles of the feet (Figure 2D, 3A). She first noted hearing loss of acute onset at 38 years of age. The paternal niece (III-5), aged 13 years, was diagnosed with bilateral mild SNHL and keratoderma on the soles of the feet (Figure 2E, 3B) and experienced hearing loss at eight years of age.

Genetic testing was conducted at the Division of Hearing and Balance Research, National Institute of Sensory Organs, and National Hospital Organization Tokyo Medical Center. In brief, genomic DNA was extracted from the blood of each participant using the Genomix kit (Biologica, Japan). The proband (III-2) was subjected to targeted resequencing of 154 known deafness genes [9]. The other family members (II-2, II-3, II-5, III-3, and III-5) were subjected to Sanger sequencing of GJB2. Genetic testing identified a heterozygous variant of GJB2, NM_004004.5: c.250G>A (p.Val84Met), in all family members with hearing loss and keratoderma (II-2, II-5, III-2, III-3, and III-5), except for one family member without these disorders (II-3). The variant was identified as “Pathogenic” according to the guidelines of the ClinGen hearing loss expert panel [10], as shown in the Appendices.

## Discussion

In this report, we present cases of a father, a son, a daughter, and their relatives affected by dominant mild SNHL carrying a heterozygous p.Val84Met variant in GJB2 with dermatological symptoms. ARNSHL 1A (DFNB1A) has been reported, with several reports associating it with the p.Val84Met variant in GJB2 [4-7]. However, the pathological significance and genetic form of the p.Val84Met variant remain undetermined [11], with no known concomitant symptoms [4-7]. In this report, we establish, for the first time, a genotype-phenotype correlation in the present family, revealing a clear association between the p.Val84Met variant, syndromic hearing loss, and keratoderma with autosomal dominant inheritance. Patients with the p.Val84Met variant reportedly experience severe-to-profound hearing loss and post-lingual hearing loss [4,7,12]. However, these findings are inconsistent with the findings from the present family. Notably, the same variant in GJB2 is associated with a variable degree of hearing loss, suggesting phenotypic variation associated with the autosomal dominant GJB2 variant. In the future, it may be necessary to identify mutant chromosomes by cytogenetic testing (karyotyping/FISH).

The function of Cx26 is impaired by GJB2 variants, which may cause either syndromic or non-syndromic hearing loss [1]. In the human epidermis, connexins play a role in keratinocyte differentiation and growth. Cx26 is frequently expressed in granular layer cells and infrequently in basal keratinocytes of the soles and palms [13]. In patients with syndromic hearing loss, the Cx26 protein variant acquires the abnormal ability to perturb skin homeostasis [13,14]. The Cx26 polypeptide includes four transmembrane domains (TM1-TM4), two extracellular domains (E1 and E2), one cytoplasmic loop (IC), and carboxyl (-COOH) and amino (-NH2) termini situated in the cytoplasm. Functional investigations have shown that dominant syndromic variants are grouped in the TM1-TM4, N-terminal, and EC1 structural domains and are absent from the C-terminal and EC2 structural domains [13,14]. The p.Val84Met variant is located in the second transmembrane domain (TM2) of GJB2. TM2 modifies the voltage-gating properties and influences the formation of functional channels [14]. In general, severe phenotypes, including keratitis ichthyosis deafness syndrome and palmoplantar keratoderma, are often caused by pathogenic variants linked to abnormal hemichannel and gap junction channel function due to dominant-negative, trans-dominant effects or cell death. Without these characteristics, pathogenic variants usually only induce a lesser form of Vohwinkel syndrome. In this case report, no severe skin diseases were observed. Therefore, the p.Val84Met variant may not be associated with dominant-negative, trans-dominant effects or cell death. However, this hypothesis should be investigated in future studies using in vitro and animal models to elucidate the mechanisms associated with the phenotypes related to the p.Val84Met variant.

In this study, a multidisciplinary team of otolaryngologists, genetic counselors, and genetic researchers played a vital role in the identification of the genetic cause of hearing loss, the inheritance pattern, and the cutaneous symptom. As the field of genetics becomes more interdisciplinary and complex, such a team of specialists is essential for adequate clinical interventions that ultimately lead to better quality of life for patients.

## Conclusions

We presented the cases of a Japanese family with autosomal dominant syndromic hearing loss (ADSHL) with keratoderma caused by c.250G>A (p.Val84Met) in GJB2. These cases establish the pathological significance of the variant and provide new insights into its genetic basis and pathological features. The variant was located in the TM2 of GJB2, suggesting an altered Cx26 channel function. A multidisciplinary team is essential for both the accurate diagnosis of and effective intervention for such diverse and complex diseases.

## Appendices

**Table 1:** Clinical interpretation of GJB2:p.Val84Met Source: [5] ^1^ In-house data indicate that seven affected segregations of the genotype with the phenotype. ^2^ Odds ratio based on our in-house data in which six heterozygotes were found among 2,201 patients with hearing loss, compared with gnomADv2.1.1 data, in which a heterozygote was found among 5,039 control Ashkenazi Jewish individuals, was 13.7 (95% confidence interval: 1.65–114, P = 0.01), which was more than 5%, and the 95% confidence interval did not contain 1. ^3^ REVEL score = 0.94. HLEP: Hearing Loss Expert Panel; OMIM: Online Mendelian Inheritance in Man; REVEL: Rare exome variant ensemble learner. This table was published under a Creative Commons License.

Title	Content
Gene	GJB2
Variant	NM_004004.5:c.250G>A (p.Val84Met)
Phenotype (OMIM)	Palmoplantar keratoderma with hearing loss (148350)
Mode of inheritance	Autosomal dominant
ClinGen HLEP Criteria	Pathogenic
Pathogenicity	PP1_Strong^1^, PS4_Strong^2^, PP3^3^
